# Two and three dimensional characterization of *Zucchini Yellow Mosaic Virus* induced structural alterations in *Cucurbita pepo* L. plants

**DOI:** 10.1016/j.jsb.2014.03.006

**Published:** 2014-05

**Authors:** Günther Zellnig, Michael Herbert Pöckl, Stefan Möstl, Bernd Zechmann

**Affiliations:** University of Graz, Institute of Plant Physiology, Schubertstrasse 51, A-8010 Graz, Austria

**Keywords:** CIs, cylindrical inclusions, dpi, days post inoculation, TEM, transmission electron microscopy, ZYMV, *Zucchini Yellow Mosaic Virus*, 3D reconstruction, Cucurbita, Cylindrical inclusions, *Zucchini Yellow Mosaic Virus*

## Abstract

Infection of plants by *Zucchini Yellow Mosaic Virus* (ZYMV) induces severe ultrastructural changes. The aim of this study was to investigate ultrastructural changes during ZYMV-infection in *Cucurbita pepo* L. plants on the two and three dimensional (2D and 3D) level and to correlate these changes with the spread of ZYMV throughout the plant by transmission electron microscopy (TEM) and image analysis.

This study revealed that after inoculation of the cotyledons ZYMV moved into roots [3 days post inoculation (dpi)], then moved upwards into the stem and apical meristem (5 dpi), then into the first true leaf (7 dpi) and could finally be found in all plant parts (9 dpi). ZYMV-infected cells contained viral inclusion bodies in the form of cylindrical inclusions (CIs). These CIs occurred in four different forms throughout the cytosol of roots and leaves: scrolls and pinwheels when cut transversely and long tubular structures and bundles of filaments when cut longitudinally. 3D reconstruction of ZYMV-infected cells containing scrolls revealed that they form long tubes throughout the cytosol. The majority has a preferred orientation and an average length and width of 3 μm and 120 nm, respectively. Image analysis revealed an increased size of cells and vacuoles (107% and 447%, respectively) in younger ZYMV-infected leaves leading to a similar ratio of cytoplasm to vacuole (about 1:1) in older and younger ZYMV-infected leaves which indicates advanced cell growth in younger tissues. The collected data advances the current knowledge about ZYMV-induced ultrastructural changes in *Cucurbita pepo*.

## 1 Introduction

*Zucchini Yellow Mosaic Virus* (ZYMV) belongs to the genus potyvirus and is one of the most destructive and widespread viral pathogens on Cucurbits worldwide. It causes severe symptoms on the leaves such as yellowing, leaf deformation, and stunting. In the later stages of infection leaves develop a yellow mosaic and often show dark green blisters (Desbiez and Lecoq, 1997; Gal-On, 2007; Lecoq and Desbiez, 2012). Fruits of ZYMV-infected plants produce less seeds, develop color alterations and deformations rendering them unmarketable (Desbiez and Lecoq, 1997).

In the sap of infected plant material ZYMV-particles can be observed after negative staining in the form of flexous, rod shaped structures with an average size between 680 and 730 nm in length and 11–13 nm in width (Gal-On, 2007; Zechmann and Zellnig, 2009). On the cellular level ZYMV induces severe ultrastructural alterations in leaves such as the appearance of proliferated endoplasmic reticulum (ER) and cylindrical inclusions (CIs) in the cytosol (Lisa et al., 1981; Lesemann et al., 1983; Petersen et al., 1991; Zechmann et al., 2003, 2005; Zechmann and Zellnig, 2009). CIs are typical ultrastructural features of viruses from the family *Potyviridae* and are formed by the potyviral CI protein. This protein is involved in cell to cell movement of the virus (Carrington et al., 1998; Roberts et al., 1998; Wei et al., 2010; Lecoq and Desbiez, 2012), in virus replication (Fernandez et al., 1995, 1997; Merits et al., 1998), and there is evidence that it interacts with other potyviral proteins such as the capsid protein (Rodriguez-Cerezo et al., 1997; Gabrenaite-Verkhovskaya et al., 2008). CIs induced by potyviruses can be categorized in four subdivisions: (1) type-1 inclusions such as pinwheels, bundles, and scrolls, (2) type-2 inclusions such as pinwheels, bundles and laminated aggregates, (3) type-3 inclusions such as pinwheels, bundles, scrolls and laminated aggregates, and (4) type-4 inclusions such as pinwheels, bundles, scrolls, and short usually curved laminated aggregates (Edwardson, 1992; Edwardson and Christie, 1996).

Even though their two dimensional (2D) ultrastructure has been described in detail, the three dimensional (3D) fine structure remains unclear. Additionally, it was unclear if CIs could also be found in roots as ultrastructural investigations in the past have mainly focused on ZYMV-infected leaves (Lisa et al., 1981; Lesemann et al., 1983; Zechmann et al., 2003, 2005; Zechmann and Zellnig, 2009). In previous studies we demonstrated that CIs induced by ZYMV in leaves did not differ between samples which were prepared by chemical fixation or cryofixation such as high pressure freezing (HPF) and plunge freezing (Zechmann et al., 2005). Nevertheless, in comparison to chemical fixed cells the overall ultrastructure was not as well preserved in plunge and high pressure frozen cells where ice crystal formation induced damages of the ultrastructure below a depth of 30–40 and 50–70 μm, respectively (Zechmann et al., 2005).

After entering leaves through wounds, cracks or injection by aphids ZYMV replicates and moves from cell to cell through plasmodesmata until it reaches sieve elements. Systemic infection is then carried out through the phloem transport system (Vuorinen et al., 2011), where virions and CIs in ZYMV-infected plants could be detected in companion cells and sieve elements (Zechmann et al., 2003, 2005). Even though the systemic spread is documented for other viruses to some extend (Wisniewski et al., 1990; Valkonen and Somersalo, 1996; Andrianifahanana et al., 1997) it still remained unclear in which time frame ZYMV spreads throughout the plant.

Besides the described ultrastructural changes within ZYMV-infected cells ZYMV and other potyviruses negatively interfere with plant metabolism such as photosynthesis (Zhou et al., 2004; Radwan et al., 2006; Spoustova et al., 2013). Related ultrastructural alterations such as the reduction in chloroplast number and thylakoid contents, and an increase in starch and plastoglobuli contents could also be resolved by quantitative transmission electron microscopy (TEM) in ZYMV-infected *Cucurbita pepo* L. plants (Zechmann et al., 2003). Nevertheless, further quantitative structural alterations (e.g., changes in the ratio of cytoplasm to vacuole) have not been investigated in further detail.

In this study the long distance spread of ZYMV was studied in *Cucurbita pepo* L. in order to investigate the time frame it needs to systemically infect its host. Additionally, the ultrastructure of pinwheels and the 3D ultrastructure of ZYMV-infected cells containing CIs in the form of scrolls was analysed by TEM and image analysis in order to get a more comprehensive view about fine structural alterations induced by ZYMV in *Cucurbita pepo* L. plants. As HPF led to dissatisfying freezing results and ice crystal damages in deeper mesophyll layers (Zechmann et al., 2005) it was impossible to perform serial sectioning with the proposed plant material in this study. Thus, we performed the present experiments with chemically fixed cells.

## 2 Material and methods

### 2.1 Plant material and ZYMV-inoculation

Seeds of *Cucurbita pepo* L. subsp. *pepo* var. *styriaca* GREB. (Saatzucht Gleisdorf Ges.mbH, Gleisdorf, Austria) were germinated in seed trays containing perlite. Seedlings were cultivated in growth chambers with a photoperiod of 12 h and a light intensity of 700 μmol m^−^^2^ s^−1^. The temperature was kept at 22 °C during the day and 20 °C during the night at a relative humidity of 60%. One week old seedlings were potted in soil and grown under the same conditions. Two weeks after germination cotyledons of one plant group were inoculated by rubbing a homogenate containing ZYMV-infected leaf material (strain id.: DSMZ PV-0466; obtained from DSMZ Plant Virus Collection, Braunschweig, Germany) and Celite (Sigma–Aldrich, Vienna, Austria) onto the cotyledons before the first foliage leaves fully emerged. The sap was obtained by homogenization of ZYMV-infected leaves in Sørensen phosphate buffer (pH 6.8). Mock inoculation was performed with control plants by rubbing Sørensen phosphate buffer without ZYMV-infected leaf material onto the cotyledons.

### 2.2 Negative staining

Negative staining was performed every 24 h as described previously (Zechmann et al., 2003, Zechmann and Zellnig, 2009). Briefly, different plant samples (see Table 1 for details) were homogenized in 0.06 M phosphate buffer (pH 6.5) and mounted on formvar coated nickel grids (400-mesh) by incubating the grids for 5 min with the obtained sap. Grids were then washed twice in buffer for 3 min each and stained with 2% phosphotungstic acid dissolved in distilled water for 2 min. Grids were then air-dried and observed by a Philips CM 10 transmission electron microscope.

## 3 Ultrastructural investigations

### 3.1 Chemical fixation

Plant material was prepared for TEM as described previously (Zechmann et al., 2007). Small pieces of roots, older and younger leaf samples (1 mm^2^) were fixed in 2.5% glutardialdehyde dissolved in 0.06 M phosphate buffer (pH 7.2) for 90 min. Samples were then rinsed in buffer (4 times 15 min) and post fixed with 1% osmium tetroxide dissolved in 0.1 M phosphate buffer (pH 7.2) for 90 min. After a rinse in buffer (4 times 10 min) specimens were dehydrated in increasing concentrations of acetone (50%, 70%, 90%, and 100%) for 2 times 10 min. Pure acetone was then exchanged with a mixture of propylene oxide and acetone (1:1) for 10 min and then finally with pure propylene oxide for 10 min. Infiltration was carried out with increasing concentrations (30%, 50%, and 70%) of Agar 100 epoxy resin (Agar Scientific) mixed with pure propylene oxide. Polymerization was performed with pure Agar 100 epoxy resin at 60 °C for 48 h. Semi-thin (4 μm) and ultra-thin sections (80 nm) were cut with a Reichert Ultracut S microtome (Leica Microsystems, Vienna, Austria), post-stained with 2% uranylic acid (5 min) dissolved in distilled water and 0.02 M lead citrate dissolved in freshly distilled water containing 0.16 M sodium hydroxide (15 min). Grids were then air-dried and observed by a Philips CM 10 and/or a Zeiss EM 902 transmission electron microscope.

### 3.2 High pressure freezing

Plant material was prepared for TEM as described previously (Zechmann et al., 2005). Briefly, small pieces (1.2 mm in diameter) of ZYMV-infected and control leaves were punched out in 1-hexadecane. The specimens were transferred onto specimen carriers and high pressure frozen in the Leica EM Pact (Leica Microsystems). For freeze substitution (FS) specimens were transferred into pre-cooled cryogenic vials (Corning Incorporated, Corning, NY 14831, USA) filled with 2% osmium tetroxide in anhydrous acetone containing 0.2% uranyl acetate. FS was carried out at −80 °C (72 h), −65 °C (24 h), −30 °C (24 h), 0 °C (12 h) and RT (1 h). After the samples were rinsed in anhydrous acetone (2 times 15 min), they were infiltrated by mixtures of acetone and Agar 100 epoxy resin (2:1, 1:1, 1:2) and finally pure epoxy resin for at least 3 h, each step. The embedded samples were then polymerised in Agar 100 epoxy resin for 48 h at 60 °C. Ultrathin sections were cut and post-stained, as described above.

### 3.3 Quantitative analysis of palisade parenchyma cells

Semi-thin sections of embedded older and younger (youngest fully developed) leaves were investigated with an Olympus Provis AX-70 (Olympus, Life and Material Science Europa GmbH, Hamburg, Germany) microscope using an UPLAN APO 40× oil immersion objective lens (Olympus, Germany). Images of the palisade parenchyma cell layer were captured with an ColorView IIIu digital camera (Olympus, Germany). The areas of the cytoplasm and the vacuole were measured using Olympus Cell F imaging software. The statistical analyses were performed by using the software package Statistica (Stat-Soft, USA, 2002). Significant differences were analyzed with Kruskal–Wallis test followed by post hoc comparison according to Conover.

### 3.4 Three dimensional reconstruction of cylindrical inclusions

Serial sections of up to 105 sections each of three individual chemically fixed samples were cut (80 nm section thickness) with a Reichert Ultracut S microtome (Leica Microsystems, Vienna, Austria). Individual sections were screened for representative cells that contained CIs in the form of scrolls throughout the cells. Digital images of every single section were taken from a selected mesophyll cell from older ZYMV-infected leaves at a primary magnification of 3.900× with a Zeiss EM 902 transmission electron microscope. Whereas CIs were reconstructed in all serial sections 3D reconstruction of other cell compartments was only performed for the most representative cell which consisted of 105 individual sections. 3D reconstruction of ZYMV-infected cells containing scrolls was performed according to Cardona et al. (2012) using the software program TrakEM2. First all images were aligned semi-automatically, then different cell structures (cell walls, chloroplasts, mitochondria, peroxisomes, vacuoles, ZYMV-induced CIs) were traced by hand on each section, and 3D reconstruction was created automatically by TrakEM2 using this information. The length of cylindrical inclusions was measured by TrakEM2 whereas the width was measured in chemically fixed and high pressure frozen samples using Olympus Cell F imaging software (Olympus, Germany) on different cell ultrathin sections.

## 4 Results and discussion

**Fig.1:**
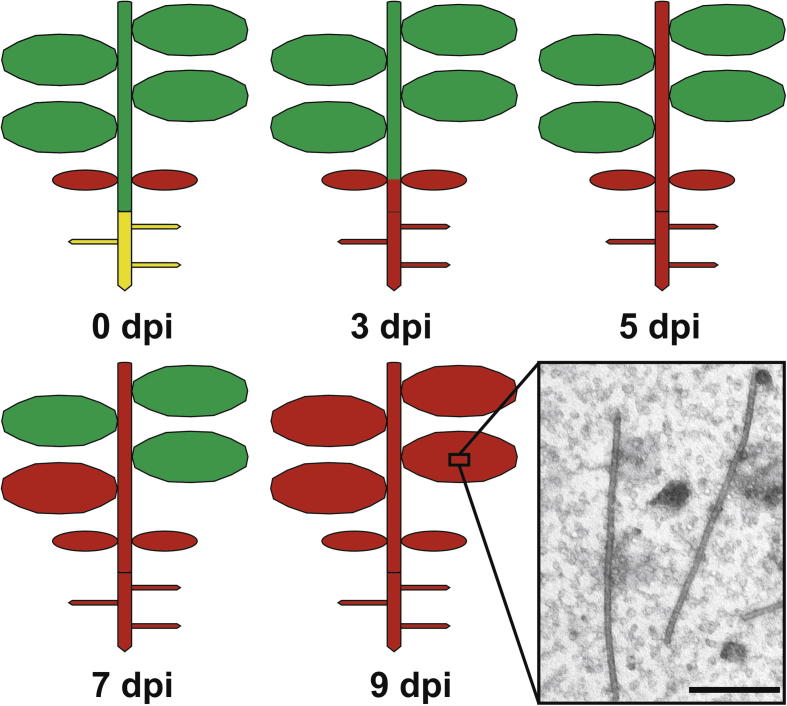
Model demonstrating the spread of ZYMV through *Cucurbita pepo* L. based on results obtained by negative staining of ZYMV-particles. After inoculation of the cotyledons ZYMV can first be detected in roots (3 dpi), then moves throughout the stem (5 dpi), and infects the first true leaves (7 dpi). Finally (9 dpi) ZYMV-particles shown in the TEM-micrograph (bar = 0.2 μm) can be detected in all plant parts by negative staining. Green = uninfected upper plant parts, red = infected plant parts, yellow = uninfected roots.

Negative staining of ZYMV-particles revealed the time course of spreading of ZYMV throughout *Cucurbita pepo* L. plants. After inoculation of the cotyledons ZYMV-particles (shown in Fig. 1) spread into roots first where they appeared 3 dpi (Table 1, Fig. 1). Virions then moved upwards and could be detected throughout the stem and in the apical meristem 5 dpi. 7 dpi ZYMV spread into first true leaves and was found throughout all parts of the plant 9 dpi (Table 1, Fig. 1). The spread of ZYMV observed in this study extends the information available in the literature for other viruses. Previous studies on systemic movement of tobacco mosaic virus (TMV) revealed that TMV coat protein could be detected within all non-inoculated leaves 6 dpi (Wisniewski et al., 1990). Coat protein of pepper mottle potyvirus was detected first 4 dpi in the internode below the inoculated leaf, then throughout the stem and finally moved in all other leaves starting at 5 dpi (Andrianifahanana et al., 1997). Symptoms of tobacco etch potyvirus could be found within 5–6 dpi in non-inoculated upper leaves (Valkonen and Somersalo, 1996). In correlation with the results from this study we can conclude that after inoculation viruses move from the inoculation site downwards towards the root and then upwards to systemically infect the whole plant. Through cell to cell movement in the form of virions and RNA strains potyviruses leave the infection site and eventually enter vascular parenchyma cells and companion cells (Vuorinen et al., 2011). The latter are responsible for loading ZYMV into the sieve tube elements where it moves in the form of virions (Zechmann et al., 2003, 2005) and systemically spreads throughout the plant (Vuorinen et al., 2011).

**Fig.2:**
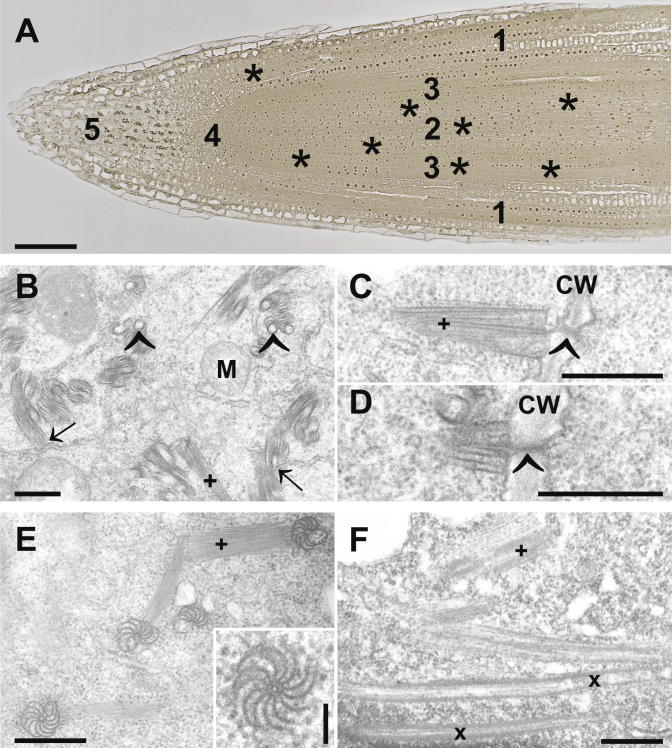
Light and electron microscopical images showing cells of roots from ZYMV-infected *Cucurbita pepo* L. plants. CIs in the form of scrolls, pinwheels and bundles of filaments could be localized in the cytosol of root cells 3 dpi at the root cortex (indicated by the number 1) and at the center and the cortex (indicated by the numbers 2 and 3, respectively) of the cell elongation zone [stars on the semithin section (A) indicate where CIs could be observed with the TEM on the following ultrathin sections] but could not been observed in the meristem or the root cap (indicated by the numbers 4 and 5, respectively) in image 2A. In root cells CIs occurred in the form of bundles of filaments (+ in B, C, E, and F) and long tubular structures (x in F) when cut longitudinally and pinwheels (inset in E) and scrolls (arrowhead in B) when cut transversely. CIs were found to be associated with the ER (arrows in B) and plasmodesmata (arrowheads in C and D). Bars = 100 μm in A, 0.5 μm in B–F, 0.1 μm in inset of E. CW = cell walls, M = mitochondria.

**Fig.3:**
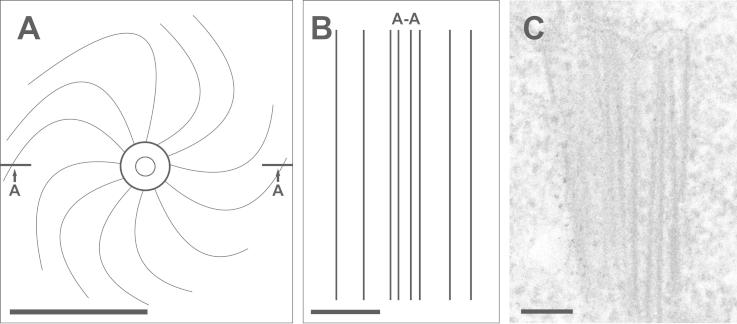
2D model (A and B) of the transversely cut pinwheel shown as inset in Fig. 2E reveals that it consists of two inner circles and 12 filaments when cut transversely (A) and as bundles of filaments if cut longitudinally (B). Image B shows cross section A-A of image A. These structures were commonly observed on fine structural level when pinwheels were cut longitudinally (C). Bars = 0.1 μm in A, C and 0.05 μm in B.

**Fig.4:**
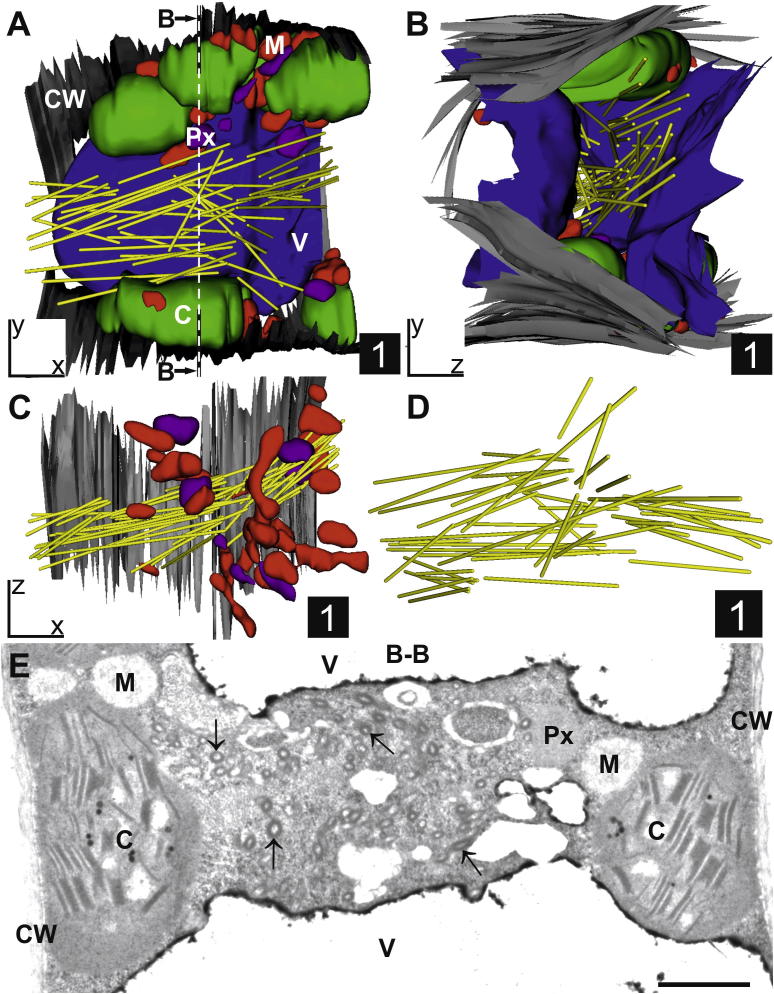
Part of a representative mesophyll cell from ZYMV-infected *Cucurbita pepo* L. leaf. 3D reconstruction of a ribbon of 105 serial sections (80 nm section thickness) demonstrating the arrangement of CIs in the form of long tubular structures (yellow tubes) throughout the cytosol from different viewing angles (A–D). The majority of these tubular structures have a preferred orientation throughout the cytosol but some differ from this direction which can be best seen when other cell structures are faded out (C and D). The rotations of the 3D reconstructions are indicated by *x*, *y*, *z* axes in the lower left corner. TEM micrograph (E) shows one representative cross section (nr. 47) indicated as section B-B in 3D reconstruction shown in image A. Cell walls (CW, black and grey structures); chloroplasts (C, green structures); mitochondria (M, red structures); peroxisomes (Px, violet structures); vacuoles (V, blue structures). Arrows in image 4E = scrolls; Square = 1 μm^2^, Bar = 1 μm.

Three dpi when virions could be detected in roots typical ultrastructural alterations such as CIs in the cytosol could be observed. They appeared in four different forms: scrolls and pinwheels when cut transversely and long tubular structures and bundles of filaments when cut longitudinally (Figs. 2B–F, 3, and 4E). According to the latest classification of potyviruses the ZYMV-strain used in this study induced cylindrical inclusions of type-1 such as pinwheels, bundles and scrolls (Edwardson, 1992; Edwardson and Christie, 1996). The observed ultrastructural alterations induced by ZYMV in roots and leaves (Figs. 2B–F, 3, and 4E) of *Cucurbita pepo* L. were similar to what was found in previous studies (Lisa et al., 1981; Lesemann et al., 1983; Zechmann et al., 2003, 2005; Zechmann and Zellnig, 2009). Through correlations of semithin sections of roots investigated by light microscopy with the following ultrathin sections investigated by TEM the occurrence of CIs in the cytosol of roots could be attributed to the root cortex and to the center and the cortex of the cell elongation zone (Fig. 2A). CIs were never found in the root cap and the root meristem. CIs were associated with the ER (Fig. 2B) and with plasmodesmata (Fig. 2C and D). The association of CIs with plasmodesmata has also been found in *Nicotiana tabacum* inoculated with the tobacco vein mottling potyvirus (Rodriguez-Cerezo et al., 1997) and in *Pisum sativum* inoculated with pea seed-borne mosaic potyvirus (Roberts et al., 1998). The authors concluded that CIs play important roles in the local transport of potyviruses from cell to cell through plasmodesmata. The ultrastructure of pinwheels found in roots (Fig. 2E) revealed that they consist of two inner circles and 12 convex, bended filaments evolving from the outer circle (Fig. 3A) when cut transversely and as 7–8 straight bundles of filaments when cut longitudinally (Fig. 3B and C). In a 3D model they would form a cylinder with 12 convex formed plates similar to what has been reconstructed in the past for wheat streak mosaic virus by analytic geometry (Mernaugh et al., 1980).

The 3D reconstruction of ZYMV-infected cells containing CIs in the form of scrolls revealed that they form long tubular structures. The majority of these tubes has a preferred orientation throughout the cytosol but some differ from this direction (Fig. 4D). The average length and width of these scrolls in chemically fixed cells was determined at 3 μm (±0.2 μm; *n* = 55 from 3 independent 3D reconstructions) and 120 nm (±5 nm; *n* = 55 from 3 independent 3D reconstructions), respectively. The width did statistically not differ from scrolls found in cells after high pressure freezing where an average width of 123 nm (±2 nm; *n* = 55 from ultrathin sections of 3 independent samples) was determined by image analysis in the present study. These results support the conclusion drawn in previous studies that the ultrastructural preservation of CIs did not differ between chemically and cryofixed cells (Zechmann et al., 2005). The length and width found in this study differed from the results obtained after studying CIs by laser scanning microscopy in ZYMV-infected *Cucurbita pepo* L. where an average length between 9.4 and 20.1 μm and an average width between 2.1 and 3.7 μm was determined depending on the time after inoculation (Lim et al., 1996). The differences between these results can be explained by the different methods used to visualize CIs. The width determined by TEM and image analysis revealed a size which is below the theoretical resolution of a light microscope (about 200 nm). In order to visualize CIs by confocal laser scanning microscopy they had to be stained with a fluorescent dye (Lim et al., 1996). Thus, the measurements reflect the fluorescence of the dye bound to CIs rather than their real size which explains the much larger width of CIs measured by confocal laser scanning microscopy. Additionally, the distance between the end and the beginning of some CIs was very small (below 200 nm), making it impossible to differentiate them from each other by fluorescence microscopy. By using TEM the fine structures of CIs can be visualized and measured directly and the obtained data are therefore closer to their real size than the data obtained in previous studies with confocal laser scanning microscopy.

**Fig.5:**
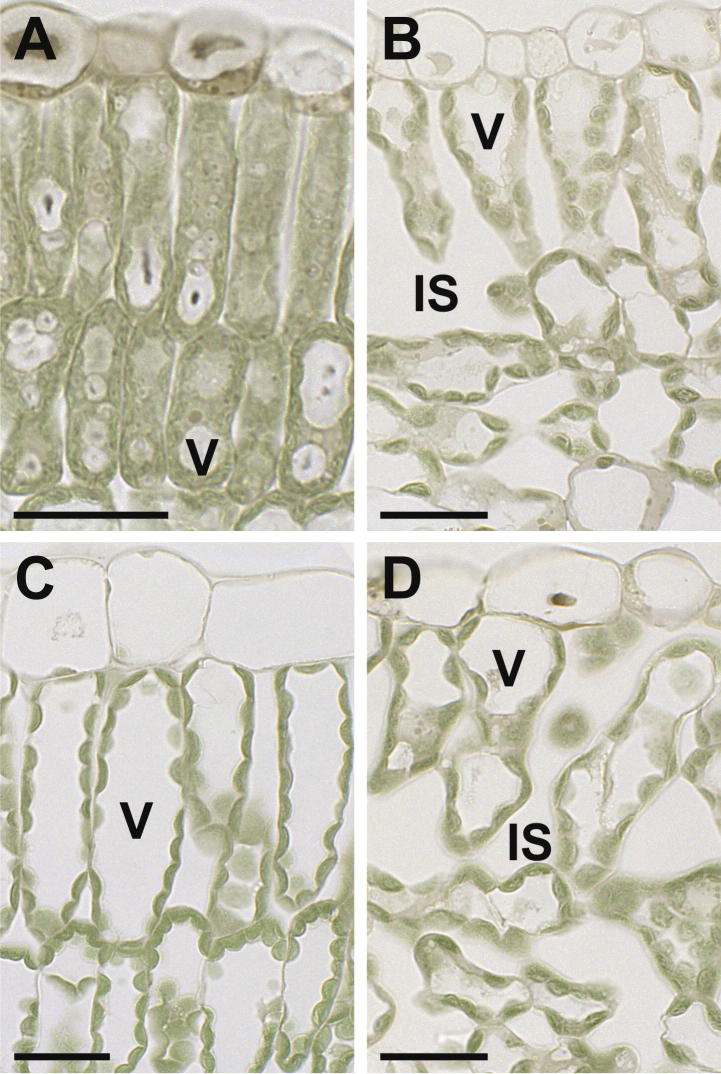
Light microscopical images showing a cross section of control (A and C) and ZYMV- infected (B and D) *Cucurbita pepo* L. leaves. Control cells from younger leaves (A) show smaller vacuoles (V) and more cytosplasm than control cells from older leaves (C). ZYMV-infected cells contain larger vacuoles in younger leaves (B) and smaller vacuoles in older leaves (D) when compared to the controls (A and C). IS = intercellular space. Bars = 25 μm.

The vacuole of cells from control older leaves generally covered a larger area (67%) of the cell than the cytoplasm (33%; Table 2, Fig. 5). These results are similar to what has been observed in other studies on Arabidopsis, peach, barley, celery and spinach where the vacuole covered between 68% and 85% of fully expanded cells (Winter et al., 1993, 1994; Nadwodnik and Lohaus, 2008; Koffler et al., 2013). A different situation was found in control younger leaves where the vacuole covered only 22% and the cytoplasm 78% of the cells. These results corroborate with the current opinion that meristematic and younger cells contain more cytoplasm than vacuoles and that the size of vacuoles increases with increasing age and developmental stage of the cell (Zouhar and Rojo, 2009). ZYMV-infection disturbed this situation as in cells of older and younger ZYMV-infected leaves about 50% were covered by vacuole and the cytoplasm (Table 2, Fig. 5). Additionally, ZYMV-infected younger leaves massively increased in cell size (107%) when compared to the control (Table 2, Fig. 5). These results indicate that ZYMV-infection induces cell growth and expansion in younger leaves leading to a similar vacuole to cytoplasm ratio in older and younger ZYMV-infected leaves. These effects might be explained by the observations that virus infections lead to the activation of senescence in plants (Espinoza et al., 2007; Stefanov et al., 2012) which could lead to a rapid aging of cells in younger ZYMV-infected leaves indicated by the strong increase in cell (107%) and vacuole size (449%) in these cells when compared to the control (Table 2, Fig. 5). It is also interesting that in older ZYMV-infected leaves an increase in cytoplasm area (plus 50%) in expense of the vacuole (minus 28%) could be observed when compared to mock inoculated control plants. These results indicate that due to metabolic changes induced by the virus a massive increase in cytoplasm occurs even though cell size remains the same between ZYMV-infected and mock inoculated older leaves (Table 2, Fig. 5).

In conclusion, ZYMV needs 3 days to move out of the inoculated leaf into roots and 9 days to systemically infect the whole plant through the phloem. Ultrastructural alterations include the appearance of CIs which can be found in the cytosol of leaves and roots and are most probably involved in the cell to cell movement through plasmodesmata. Additionally, ZYMV-infection led to advanced cell growth and development in younger leaves and increased the area of the cytoplasm in older ones.

Footnote: CIs found in this study appear in four different forms (scrolls, pinwheels, tubes and bundles). The term CIs is used where no distinction was drawn between these forms by us or other authors and where all forms may be concerned.

## Figures and Tables

**Table 1:** Spread of ZYMV through different parts of *Cucurbita pepo* L. based on results obtained by negative staining. Sampling was done every 24 h after inoculation.

dpi	sp 1	sp 2	sp 3	sp 4	sp 5	sp 6	sp 7	sp 8	sp 9	sp 10
1										
2										
3	+									
4	+									
5	+	+		+		+		+		+
6	+	+		+		+		+		+
7	+	+	+	+		+		+		+
8	+	+	+	+		+		+		+
9	+	+	+	+	+	+	+	+	+	+

days post inoculation (dpi), (+) indicates virion appearance, sampling points (sp): sp 1 = root tips, sp 2 = Epicotyl, sp 3 = leaf #1, sp 4 = first internode, sp 5 = leaf #2, sp 6 = second internode, sp 7 = leaf #3, sp 8 = third internode, sp 9 = leaf #4, sp 10 = apical meristematic zone.

**Table 2:** Data are means with standard errors and document the relative palisade parenchyma cell area without cell walls (in μm^2^) and the percentage of total cell area of the cytoplasm and vacuoles measured on longitudinal semi-thin sections of younger (YL) and older (OL) ZYMV-infected and control leaves. Significant differences among samples of one cell structure are indicated by different lowercase letters. *P* < 0.05 was regarded significant analyzed with Kruskal–Wallis test followed by post hoc comparison according to Conover. *n* = number of cells.

	*n*	Total cell area (μm^2^)	Cytoplasm (μm^2^)	% of total cell area	Vacuole (μm^2^)	% of total cell area
Control YL	182	333 ± 5^c^	260 ± 5^d^	78 ± 0.6^a^	73 ± 3^d^	22 ± 0.6^c^
Virus YL	148	688 ± 18^b^	360 ± 7^b^	52 ± 1^b^	328 ± 11^c^	48 ± 0.8^b^
Control OL	112	912 ± 26^a^	300 ± 7^c^	33 ± 0.8^c^	612 ± 23^a^	67 ± 0.8^a^
Virus OL	131	888 ± 44^a^	450 ± 20^a^	51 ± 1.2^b^	439 ± 30^b^	49 ± 1.2^b^

## References

[b0005] Andrianifahanana M., Lovins K., Dute R., Sikora E., Murphy J.F. (1997). Pathway for phloem-dependent movement of pepper mottle potyvirus in the stem of *Capsicum annum*. Virology.

[b0010] Cardona A., Saalfeld S., Schindelin J., Arganda-Carreras I., Preibisch S., Longair M., Tomancak P., Hartenstein V., Douglas R.J. (2012). TrakEM2 software for neural circuit reconstruction. PLoS One.

[b0015] Carrington J.C., Jensen P.E., Schaad M.C. (1998). Genetic evidence for an essential role for potyvirus CI protein in cell-to-cell movement. Plant J..

[b0020] Desbiez C., Lecoq H. (1997). Zucchini yellow mosaic virus. Plant Pathol..

[b0025] Edwardson J.R. (1992). Inclusion bodies. Arch. Virol. Suppl..

[b0030] Edwardson J.R., Christie R.G. (1996). Cylindrical inclusions. Florida Agricultural Experimental Station bulletin no. 894.

[b0035] Espinoza C., Medina C., Somerville S., Arce-Johnson P. (2007). Senescence-associated genes induced during compatible viral interactions with grapevine and *Arabidopsis*. J. Exp. Bot..

[b0040] Fernandez A., Lain S., Garcia J.A. (1995). RNA helicase activity of the plum pox potyvirus CI protein expressed in *Escherichia coli*. Mapping of an RNA binding domain. Nucleic Acids Res..

[b0045] Fernandez A., Guo H.S., Saenz P., Simon-Buela L., Gomez de Cedron M., Garcia J.A. (1997). The motif V of plum pox potyvirus CI RNA helicase is involved in NTP hydrolysis and is essential for virus RNA replication. Nucleic Acids Res..

[b0050] Gabrenaite-Verkhovskaya R., Andreev I.A., Kalinina N.O., Torrance L., Taliansky M.E., Mäkinen K. (2008). Cylindrical inclusion protein of potato virus A is associated with a subpopulation of particles isolated from infected plants. J. Gen. Virol..

[b0055] Gal-On A. (2007). Zucchini yellow mosaic virus: insect transmission and pathogenicity-the tails of two proteins. Mol. Plant Pathol..

[b0065] Koffler B.E., Bloem E., Zellnig G., Zechmann B. (2013). High resolution imaging of sub-cellular glutathione concentrations by quantitative immunoelectron microscopy in different leaf areas of *Arabidopsis*. Micron.

[b0070] Lecoq H., Desbiez C. (2012). Viruses of cucurbit crops in the mediterranean region: an ever-changing picture. Adv. Virus Res..

[b0075] Lesemann D.E., Makkouk K.M., König R., Samman E.N. (1983). Natural infection of cucumbers by zucchini yellow mosaic virus in Lebanon. Phytopathol. Z..

[b0080] Lim T.M., Chng C.G., Wong S.M. (1996). Study of the three-dimensional images of potyvirus induced cytoplasmic inclusions using confocal laser scanning microscopy. J. Virol. Meth..

[b0085] Lisa V., Boccardo G., D’Agostino G., Dellavalle G., D’Aquilio M. (1981). Characterization of a potyvirus that causes zucchini yellow mosaic. Phytopathology.

[b0090] Merits A., Guo D.Y., Saarma M. (1998). VPg, coat protein and five non-structural proteins of potato A potyvirus bind RNA in a sequence-unspecific manner. J. Gen. Virol..

[b0095] Mernaugh R.L., Gardner W.S., Yocom K.L. (1980). Three-dimensional structure of pinwheel inclusions as determined by analytic geometry. Virology.

[b0100] Nadwodnik J., Lohaus G. (2008). Subcellular concentrations of sugar alcohols and sugars in relation to phloem translocation in *Plantago major*, *Plantago maritima*, *Prunus persica*, and *Apium graveolens*. Planta.

[b0105] Petersen M.A., Edwardson J.R., Lecoq H., Purcifull D.E. (1991). Morphological variation of inclusions induced by zucchini yellow mosaic virus isolates. Phytopathology.

[b0110] Radwan D.E.M., All Fayez K., Mahmoud Y., Hamad A., Lu G. (2006). Salicylic acid alleviates growth inhibition and oxidative stress caused by zucchini yellow mosaic virus infection in Cucurbita pepo leaves. Physiol. Mol. Plant Pathol..

[b0115] Roberts I.M., Wang D., Findlay K., Maule A.J. (1998). Ultrastructural and temporal observations of the potyvirus cylindrical inclusions (CIs) show that the CI protein acts transiently in aiding virus movement. Virology.

[b0120] Rodriguez-Cerezo E., Findlay K., Shaw J.G., Lomonossoff G.P., Qui S.G., Linstead P., Shanks M., Risco C. (1997). The coat and cylindrical inclusion proteins of a potyvirus are associated with connections between plant cells. Virology.

[b0125] Spoustova P., Synkova H., Valcke R., Cerovska N. (2013). Chlorophyll a fluorescence as a tool for a study of the Potato virus Y effects on photosynthesis of nontransgenic and transgenic Pssu-ipt tobacco. Photosynthetica.

[b0130] Stefanov D., Stoimenova E., Marinova G., Ivanova B., Edreva A. (2012). Accelerated leaf senescence takes part in enhanced resistance in cucumber mosaic virus inoculated pepper leaves. Acta Physiol. Plant..

[b0135] Valkonen J.P.T., Somersalo S. (1996). Patterns and barriers of cell-to-cell movement and lack of systemic spread of tobacco etch potyvirus (TEV-GUS) in *Solanum brevidens*. Plant Sci..

[b0140] Vuorinen A.L., Kelloniemi J., Valkonen J.P.T. (2011). Why do viruses need phloem for sistemi invasion of plants?. Plant Sci..

[b0145] Wei T., Zhang C., Hong J., Xiong R., Kasschau K.D., Zhou X., Carrington J.C., Wang A. (2010). Formation of complexes at plasmodesmata for potyvirus intercellular movement is mediated by the viral protein P3N-PIPO. PLoS Pathog..

[b0150] Winter H., Robinson D.G., Heldt H.W. (1993). Subcellular volumes and metabolite concentrations in barley leaves. Planta.

[b0155] Winter H., Robinson D.G., Heldt H.W. (1994). Subcellular volumes and metabolite concentrations in spinach leaves. Planta.

[b0160] Wisniewski L.A., Powell P.A., Nelson R.S., Beachy R.N. (1990). Local and systemic spread of tobacco mosaic virus in transgenic tobacco. Plant Cell.

[b0165] Zechmann B., Müller M., Zellnig G. (2003). Cytological modifications in zucchini yellow mosaic virus (ZYMV)-infected Styrian pumpkin plants. Arch. Virol..

[b0170] Zechmann B., Müller M., Zellnig G. (2005). Effects of different fixation and freeze substitution methods on the ultrastructural preservation of ZYMV-infected *Cucurbita pepo* (L.) leaves. J. Electron Microsc..

[b0175] Zechmann B., Müller M., Zellnig G. (2007). Membrane associated qualitative differences in cell ultrastructure of chemically and high pressure cryofixed plant cells. J. Struct. Biol..

[b0180] Zechmann B., Zellnig G. (2009). Rapid TEM diagnosis of plant virus diseases. J. Virol. Methods.

[b0185] Zhou Y.H., Peng Y.H., Lei J.L., Zou L.Y., Zheng J.H., Yu J.Q. (2004). Effects of potato virus Y ^NTN^ infection on gas exchange and photosystem 2 function in leaves of *Solanum tuberosum* L.. Photosynthetica.

[b0190] Zouhar J., Rojo E. (2009). Plant vacuoles: where did they come from and where are they heading?. Curr. Opin. Plant Biol..

